# SFQMamba: A Spatial–Frequency Deraining Framework for Robust Visual Sensing in UAV-Assisted IoT Systems

**DOI:** 10.3390/s26123680

**Published:** 2026-06-09

**Authors:** Letian Deng, Chunyu Meng, Yuhong Zhou, Yuechao Guo, Zhiming Guo, Di Ya, Jianhai Yang, Huaibo Song, Lifeng Qin

**Affiliations:** 1 College of Mechanical and Electronic Engineering, Northwest A&F University, Yangling, Xianyang 712100, China; 2001121066@nwafu.edu.cn (L.D.); 3409361293@nwafu.edu.cn (C.M.); 2025056111@nwafu.edu.cn (Y.Z.); 2021012582@nwafu.edu.cn (Y.G.); guozhiming1999@nwafu.edu.cn (Z.G.); 2025056102@nwafu.edu.cn (D.Y.); 2025056106@nwafu.edu.cn (J.Y.); songhuaibo@nwsuaf.edu.cn (H.S.); 2 Key Laboratory of Agricultural Internet of Things, Ministry of Agriculture and Rural Affairs, Yangling, Xianyang 712100, China; 3 Shaanxi Key Laboratory of Agricultural Information Perception and Intelligent Service, Yangling, Xianyang 712100, China

**Keywords:** dual-branch architecture, UAV, CNN, Mamba, image deraining

## Abstract

Existing single-image deraining methods often exhibit limited 2D long-range dependency modeling and underexploit frequency-domain priors. To address this, we propose SFQMamba, a dual-branch deraining network based on spatial–frequency feature fusion. The CNN branch employs a Fused Enhance Block (FEB), which integrates multi-scale spatial modeling with global frequency modulation, supported by residual coupling and channel guidance, to suppress rain streaks and recover structural details. Concurrently, the Mamba branch utilizes a Spatial-Aware Selective Fusion Block (SASFB). By incorporating a four-directional scanning mechanism and adaptive path-gating, SASFB extends 1D State Space Models into the 2D domain for content-aware feature fusion. Features from both branches are hierarchically aggregated via concatenation and pointwise convolution. Experiments on the Rain13K and Raindrop datasets show that SFQMamba provides robust restoration. Compared with TransMamba, it obtains improvements of 0.12 dB in PSNR and 0.11% in SSIM, removing dense rain streaks while preserving structural and textural details. Furthermore, on the RainVisDrone benchmark, specifically the medium-rain subset, our method improves YOLOv8s detection by 0.0737 AP, 0.1060 AP${⁠}_{50}$, and 0.0897 AP${⁠}_{75}$ over degraded inputs. These results indicate that the proposed framework benefits both low-level visual restoration and downstream object perception in UAV applications.

## 1. Introduction

In complex outdoor scenarios, rain acts as a widespread atmospheric degradation that severely compromises image quality, leading to loss of background details and a reduction in local contrast [1,2,3]. This type of degradation not only affects human visual perception, but also poses significant challenges to the stability of subsequent computer vision systems, including autonomous driving [4,5], video surveillance [6,7], drone remote sensing, and object detection [8,9]. In UAV-assisted IoT applications, the problem of poor image quality caused by rainy weather is particularly important. This is because the visual data captured by UAVs is often transmitted to edge devices or ground stations for real-time perception, monitoring, and decision-making. If rain streaks block the edges of objects and weaken local contrast, the transmitted visual data will not be as reliable for subsequent recognition and IoT services that value security. Therefore, Single Image Deraining (SID) remains a highly active area of research, where effective decoupling of rain streaks from background details remains a significant hurdle [10,11,12].

In UAV-assisted Internet of Things (IoT) systems, visual data acquired by aerial platforms are frequently transmitted to edge servers, ground stations, or distributed sensing nodes for real-time monitoring, target detection, environmental perception, and emergency response. However, in rainy weather, degraded visual observations can reduce the reliability of sensing data before they are encoded, transmitted, and interpreted by downstream IoT applications [13,14]. From the perspective of secure and reliable UAV-IoT communication, the quality and integrity of the visual source data are as critical as the protection of the communication link itself. Although existing studies on UAV-enabled secure communications mainly focus on authentication, secrecy, anti-jamming, privacy preservation, and resource optimization, adverse-weather visual degradation remains an under-explored factor that can weaken the trustworthiness of perception-driven IoT services [15,16,17]. Consequently, developing an efficient deraining model for UAV imagery is meaningful not only for low-level image restoration, but also for robust visual sensing and reliable information acquisition in UAV-assisted IoT systems [18,19,20]. Compared with many close-range scenes, UAV images often contain wide-view backgrounds, small objects, large scale variations, and rain streaks with diverse orientations. These characteristics require a deraining model to preserve local object details while capturing long-range structural dependencies.

Early deraining methods relied mainly on physical priors and handcrafted constraints, such as sparse coding [2], low-rank models [21], and Gaussian Mixture Models (GMMs) [22]. However, these methods often require complex parameter tuning and exhibit limited generalization across diverse real-world rainy conditions due to their limited capacity to handle multi-scale rain streaks [23,24].

Recently, deep learning has significantly advanced image deraining, and existing methods can be broadly divided into Convolutional Neural Network (CNN)-based [10,25,26] and Transformer-based [27,28,29] approaches. CNN-based methods are effective in local texture restoration owing to their inherent local inductive bias [30,31]. Ren et al. [26] developed a progressive recurrent network called PReNet, which expands the shallow ResNet and improves its performance on both synthetic and real-world datasets. Zamir et al. [32] proposed a multi-stage progressive repair method, also known as MPRNet, which optimizes degraded inputs step by step through supervision at each stage. However, the locality of convolution restricts the receptive field, making it difficult to model long-range spatial dependencies, particularly for elongated rain streaks distributed across large image regions.

To address these shortcomings, Transformer-based methods alleviate this limitation by using self-attention to capture global contextual information [33]. Restormer [27] achieved state-of-the-art performance in tasks such as rain removal and deblurring by promoting the interaction between long-distance pixels. Nevertheless, conventional self-attention incurs quadratic computational complexity with respect to image resolution, which limits its efficiency in high-resolution restoration tasks [34,35,36,37].

Structured State Space Models (SSMs), such as S4 [38] and its derivative Mamba [39], have recently emerged as efficient alternatives for long-range dependency modeling. By combining the linear complexity of RNNs with the global modeling capability of Transformers, Mamba can maintain good computational efficiency and make significant improvements in the capture of long-range dependencies [40]. Standard state space models are generally time invariant, with fixed parameters, which makes it difficult for them to adapt well to different content for modeling. To address this shortcoming, the Mamba architecture has added a selection mechanism that allows the model parameters to follow the input data. Mamba can achieve linear complexity of the O(N) level for selective scanning algorithms with hardware perception. Subsequent models such as Vision Mamba [41] demonstrate the possibility of bidirectional SSM replacing ViT in high-resolution visual tasks.

In research related to image restoration, methods developed using the Mamba architecture have gradually gained more attention recently. Sun et al. [42] proposed a hybrid Transformer Mamba network for image rain removal tasks in 2026, which uses cascaded bidirectional SSM to enhance the modeling effect of long-range features. Recent Mamba-based image restoration methods have further explored the potential of state space modeling in low-level vision. FourierMamba [43] applies state space modeling in the Fourier domain to capture frequency correlations associated with rain degradation. TAMambaIR [44] has developed a state space model that is aware of texture for image restoration and added a rain removal effect evaluation step. These studies demonstrate that Mamba-based correlation modeling can effectively support image restoration tasks.

Despite these advances, currently using existing methods such as Mamba for rain removal and image restoration still has limitations. Directional scanning methods struggle to identify all raindrop patterns in every direction within two-dimensional images, and the fusion of feature information may lead to feature redundancy and conflicts. To address these issues, we constructed a two-branch spatial–frequency framework and incorporate four-directional selective modeling capabilities.

**Figure 1:**
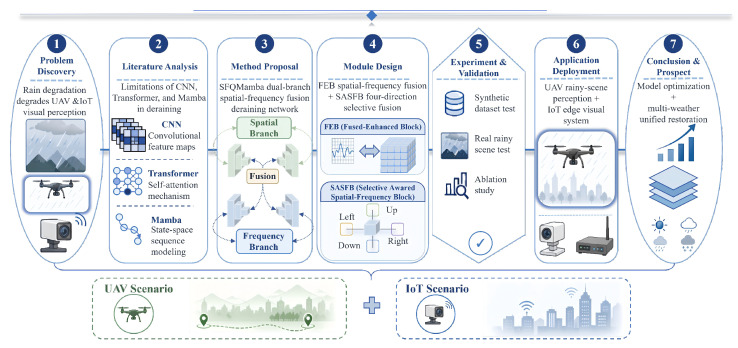
Overall framework of the proposed research workflow.

Figure 1 illustrates the overall research workflow. We noticed that after the rainfall situation becomes more severe, the visual perception of drones and IoT scenarios will be greatly affected, and image quality is particularly critical in subsequent recognition, monitoring, and edge intelligence tasks. After sorting out the shortcomings of current methods for rain removal using CNN, Transformer, and Mamba, we proposed the SFQMamba dual branch spatial frequency fusion network for single image rain removal. The FEB module combines local multi-scale spatial modeling with global frequency domain modulation to enhance the representation of spatial frequencies. The SASFB module introduces a four directional selective modeling approach to capture the long-range dependencies of rain streaks in different directions. We validate the proposed framework through comprehensive benchmarks and ablation studies. We can use the expected recovery results to help improve the rain perception ability of drones in adverse weather conditions, as well as the edge vision system on the Internet of Things side. The proposed two-branch algorithm for networks comprises the following two aspects:

- Fused Enhance Block (FEB): The spatial distribution of rain patterns is irregular, but they have quasi periodic characteristics in the frequency domain. We designed FEB to adjust the spectral amplitude as a whole in the frequency domain, which can correct structural distortion problems. In the spatial domain, multi-scale convolution is used to capture local details, and a learnable residual coupling strategy is also added for adaptive spatial frequency fusion. In this way, the model’s perception ability of the image structure can be strengthened in complex rainy scenes.
- Spatial-Aware Selective Fusion Block (SASFB): To address the shortcomings of the existing 2D-SSM architecture in directional modeling and to solve its redundancy fusion problem, we have designed a spatially aware selective fusion block, namely SASFB. We used a four directional selective scanning method to expand the original one-dimensional SSM into two-dimensional space, and then combine it with an adaptive path gating mechanism to selectively fuse horizontal and vertical features as needed. This approach can maintain stability during the training process and enable the model to better cope with multi-directional rain pattern interference.

Experimental results demonstrate that SFQMamba achieves competitive and stable performance compared to representative deraining methods, including recent Mamba-related baselines such as TransMamba [42], FourierMamba [43], and TAMambaIR [44], on synthetic rain benchmarks. To further verify the application value, we perform a UAV rainy-scene object-detection evaluation on VisDrone-derived RainVisDrone data [45], and the results show that the proposed deraining model improves target detection performance under rainy conditions. It should be clarified that this work is not the first study to address UAV-specific image degradation or accuracy problems. SFQMamba is proposed as a general single-image deraining framework, and the UAV-oriented evaluation is used to verify its potential value as a visual enhancement front-end for rainy UAV-IoT perception scenarios.

## 2. Method

### 2.1. Overall Network Architecture

**Figure 2:**
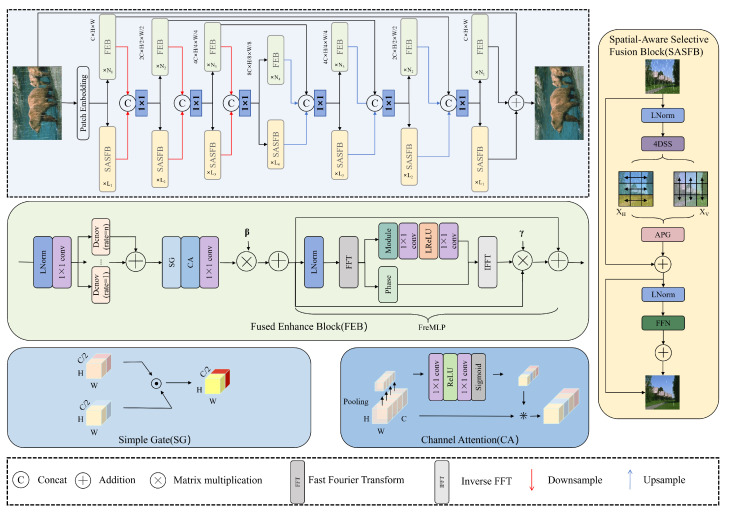
Overall architecture of the proposed SFQMamba. In the figure, ⊙ denotes element-wise multiplication, whereas * denotes channel-wise multiplication.

To reconcile the trade-off between global dependency modeling and local detail preservation, we propose SFQMamba (Spatial–Frequency QuadMamba). As shown in Figure 2, the network adopts a symmetric hierarchical encoder–decoder architecture. The input image is first processed by patch embedding for initial feature partitioning and representation mapping. The extracted features are then fed into a dual-stream collaborative framework consisting of a Mamba branch and a spatial–frequency branch.

The Mamba branch employs multiple Spatial-Aware Selective Fusion Blocks (SASFB) to capture omnidirectional long-range dependencies. In parallel, the spatial–frequency branch introduces Fused Enhance Blocks (FEB) to preserve local details and provide complementary frequency-domain information. At each hierarchical level, features from the two branches are integrated by concatenation followed by pointwise convolution, enabling effective interaction between global contextual representations and local spatial–frequency features.

As indicated by the tensor dimensions in Figure 2, the encoder progressively reduces the spatial resolution while increasing the channel capacity, while the decoder performs the reverse process to recover high-resolution representations. Pixel Unshuffle [46] and Pixel Shuffle [47] are adopted for downsampling and upsampling, respectively. Unlike pooling or transposed convolution, these operations rearrange spatial pixels and therefore help preserve feature information during resolution transformation. In addition, long-range skip connections bridge the encoder and decoder, providing fine-grained structural cues for the final deraining reconstruction.

The detailed structures of the Simple Gate (SG) and Channel Attention (CA) modules are also presented in Figure 2.

Although no handcrafted UAV-specific prior is explicitly embedded in the network, the design of SFQMamba is aligned with the characteristics of UAV rainy images. The spatial–frequency branch helps to recover the boundaries of small objects and local textures, while the Mamba branch models long-range rain-streak dependencies in wide-view aerial scenes. Thus, the proposed framework remains general for image deraining, but its design is well suited to UAV-oriented visual sensing.

### 2.2. Fused Enhance Block

#### 2.2.1. Spatial Modulation Enhancement

In rainy images, the rain streaks are far from uniform. Their thickness, length, and orientation may change significantly across different regions, making it difficult for a convolution with a single fixed receptive field to describe all rain patterns effectively. This limitation becomes more evident when thin streaks and long directional rain structures appear simultaneously. For this reason, the spatial branch adopts a multi-scale dilated convolution design to capture rain-related structures over different spatial ranges. For an input feature $X_{in}\in R^{H\times W\times C}$, where R denotes the space of real-valued tensors, *H* and *W* represent the spatial dimensions corresponding to the height and width of the feature map, and *C* denotes the channel dimension that encodes different responses to features. Layer normalization is applied before feature transformation to reduce distribution fluctuation. A 1×1 convolution is then used to expand the channel representation, providing a richer feature space for subsequent spatial modeling. The expanded features are further processed by three parallel depthwise separable convolutions with dilation rates of 1, 4 and 9, so that local details, medium-scale rain patterns, and longer directional streaks can be extracted in a unified structure. This multi-scale design approach allows the network to simultaneously capture local texture details and long-distance spatial dependencies without increasing computational complexity. The formula for multi-scale feature fusion is derived and expressed as follows:

(1) $$Z_{\mathrm{branches}}=\sum_{d\in \{1,4,9\}}{\mathrm{DWConv}}_{3\times 3}^{\left(d\right)}\;\left(\mathrm{Proj}\;\left(\mathrm{LN}(X_{\mathrm{in}})\right)\right)$$

In this architecture, LN [48] refers to layer normalization, while Proj represents the 1×1 feature projection operation. To ensure spatial consistency between feature maps, a 3×3 deep convolution is used in each branch, and the size of the padding is dynamically adjusted according to the expansion rate *d* [49]. The combination of different expansion rates can enable the model to more efficiently expand the receptive field, so that the network can capture all multi-scale features, from fine local details to wide global contexts, without the usual increase in computational costs with more parameters. It is quite difficult to distinguish between effective signals and background interference, so we also added a nonlinear gating mechanism called SimpleGate (SG) [50] to strictly suppress noise and separate obvious features. We illustrate the mechanism in Figure 2, which breaks down features along the dimension of the channel and facilitates adaptive element wise multiplication, effectively filtering out useless redundant information. We used the Channel Attention (CA) module [51] in this step, which can help adjust the weights of the feature channels and complement the previous filtering operations. This can make the network more sensitive to residual rain streaks, and the model will focus its attention on the areas most severely damaged. Finally, a 1×1 convolution will be used to project and synthesize the processed signals, resulting in a spatially enhanced feature map $Z_{spatial}$, which contains a lot of multi-scale contextual information.

(2) $$Z_{spatial}={\mathrm{Conv}}_{1\times 1}\left(\mathrm{CA}\left(\mathrm{SimpleGate}\left(Z_{branches}\right)\right)\right)$$

This spatial path can securely extract enough contextual clues, while also preserving subtle differences and semantic information within the region.

To prevent spatial enhancement from excessively altering the representation of the backbone, spatial output is further introduced through a learnable residual coupling coefficient β. Instead of directly adding the enhanced feature to the input, β adaptively scales the contribution of $Z_{spatial}$ before residual injection, stabilizing gradient propagation and allowing the network to emphasize rain-degraded structures while preserving the integrity of the original representation. The resulting residual-coupled spatial representation is defined in Equation (3) and subsequently passes through the frequency pathway.

(3) $$Y=X_{in}+\beta \cdot Z_{spatial}$$

#### 2.2.2. Frequency Modulation Enhancement

Although multi-scale convolutions are effective in extracting local textures, they are limited in modeling global structural consistency and suppressing frequency-specific rain artifacts. To complement spatial representations, we introduce a frequency-domain modulation pathway based on the Fourier Transform.

Given the spatially enhanced feature $Y\in R^{H\times W\times C}$, Layer Normalization is first applied, and then a two-dimensional FFT is performed independently on each channel over the spatial dimensions H×W:

(4) $$F\left(Y\right)={\mathrm{FFT}}_{2D}\left(\mathrm{LN}\left(Y\right)\right).$$

Here, $F\left(Y\right)\in C^{H\times W\times C}$ denotes the complex-valued frequency-domain representation. Its amplitude spectrum and phase spectrum are denoted by $A_{Y}$ and $P_{Y}$, respectively. In our implementation, FreMLP does not directly process the complex-valued tensor. Instead, it is applied only to the real-valued amplitude spectrum $A_{Y}$ to modulate spectral magnitudes, while the phase spectrum $P_{Y}$ is preserved to maintain the spatial structure. The frequency-enhanced feature is reconstructed by

(5) $$X_{\mathrm{freq}}={\mathrm{IFFT}}_{2D}\left(\mathrm{FreMLP}\left(A_{Y}\right)⊙\exp\left(jP_{Y}\right)\right),$$

where ${\mathrm{IFFT}}_{2D}$ denotes the two-dimensional inverse Fourier transform, *j* is the imaginary unit, and ⊙ represents element-wise multiplication.

Through this amplitude-modulation strategy, FreMLP suppresses abnormal frequency responses caused by repetitive rain streaks without introducing complex-valued network operations. The retained phase information helps preserve the structural layout during frequency-domain reconstruction.

After spectral reconstruction, $X_{\mathrm{freq}}$ serves as a global spectral guidance signal to modulate spatial representation.

(6) $$X_{\mathrm{out}}=Y+\gamma \cdot \left(Y⊙X_{\mathrm{freq}}\right),$$

where γ is a learnable scaling coefficient. This multiplicative guidance enables FEB to selectively enhance or suppress spatial responses according to global frequency cues, improving its adaptability to rain streaks with different densities and orientations.

In general, FEB integrates β-guided spatial enhancement and γ-guided frequency modulation to jointly preserve local details and correct global structures, thereby improving robustness to complex rain patterns.

### 2.3. Spatial-Aware Selective Fusion Block

State space models were originally developed for one-dimensional sequence modeling. To apply it to two-dimensional images, it usually requires limited scanning methods, such as horizontal scanning, vertical scanning, or bidirectional traversal. However, such scanning schemes may lead to uneven spatial coverage and insufficient modeling of diagonal or cross-axis long-range dependencies. These limitations are particularly evident in the task of removing rain streaks from images, as the angles of the streaks are random and traditional one-dimensional scanning methods cannot account for all possible scenarios. In addition, the commonly used method for combining the features of different scanning axes using basic summation or concatenation cannot flexibly adapt to the geometric changes in the rain streaks.

To address these limitations, we have developed a spatial perception selective fusion block, also known as SASFB, which relies on four-way selective scanning, also known as 4DSS, to ensure that the context in the two-dimensional space is fully captured. We use adaptive path gating, also known as the APG mechanism, to dynamically synthesize features from different scanning axes, which can significantly improve the model’s robustness against rain streaks in any direction.

#### 2.3.1. Four-Directional Selective Scan

**Figure 3:**
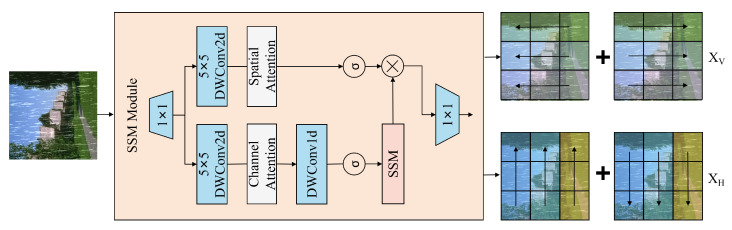
Illustration of the 4DSS module.

The 4DSS mechanism, as the SASFB core feature extraction engine, expands the receptive field of the SSM and can cover almost all major directions in the 2D plane. Taking the situation in Figure 3 as an example, we constructed four non-interfering SSM paths, scanning the entire feature map one by one along both the horizontal and vertical directions.

The input feature *X* is in the format of $R^{B\times C\times H\times W}$, where *B* stands for batch size, which refers to the number of data samples processed together in one forward pass through the neural network. At the beginning of the entire process, a normalization layer, namely LN, is performed, and finally the tensor $F_{n}$ is obtained. Afterwards, this standardized tensor will be sent to the SSM module. We refer to the analysis framework established in [39] and formalize the algorithm execution process of the SSM module.

(7) $$F_{1},F_{2}={\mathrm{Conv}}_{1\times 1}\left(F_{n}\right)$$

(8) $$F_{3}=\mathrm{SSM}\left(\sigma \left(\mathrm{Conv}1d\left(\mathrm{SA}\left({\mathrm{Conv}}_{5\times 5}\left(F_{1}\right)\right)\right)\right)\right)$$

(9) $$F_{4}=F_{1}\cdot \sigma \left(\mathrm{CA}\left({\mathrm{Conv}}_{5\times 5}\left(F_{2}\right)\right)\right)$$

(10) $$F_{5}={\mathrm{Conv}}_{1\times 1}\left(F_{4}\right)$$

Normalized features are projected into the expanded feature space. After projection, split it into two branches along the direction of the channel, which are called $F_{1}$ and $F_{2}$, respectively. The spatial attention branch processes $F_{1}$ to generate the corresponding spatial attention maps, which are used to identify areas that are more sensitive to changes in rainfall. The channel attention branch will use $F_{2}$ to calculate the response weights for each channel. Then, using these two attention results, adjust the characteristic $F_{3}$ extracted from SSM to obtain the modulated characteristic $F_{4}$. Finally, perform projection processing on $F_{4}$ to obtain the final $F_{5}$.

We aggregate information acquired from four scanning directions and classify it into two axial feature representations: horizontal feature $X_{H}$ and vertical feature $X_{V}$. Specifically, $X_{H}$ fuse results from bidirectional horizontal scanning, while $X_{V}$ handles components along the vertical orientation. This approach of doing things separately not only allows the model to follow the main axis direction and fully model the bidirectional long-distance dependencies, but also produces many special axial features, which can help with adaptive fusion. The mathematical formulation for these axial features is given by:

(11) $$X_{H}={\mathrm{Mamba}}_{LR}\left(\mathrm{LN}\left(X\right)\right)+{\mathrm{Mamba}}_{RL}\left(\mathrm{LN}\left(X\right)\right)$$

(12) $$X_{V}={\mathrm{Mamba}}_{TB}\left(\mathrm{LN}\left(X\right)\right)+{\mathrm{Mamba}}_{BT}\left(\mathrm{LN}\left(X\right)\right)$$

#### 2.3.2. Adaptive Path Gate

**Figure 4:**
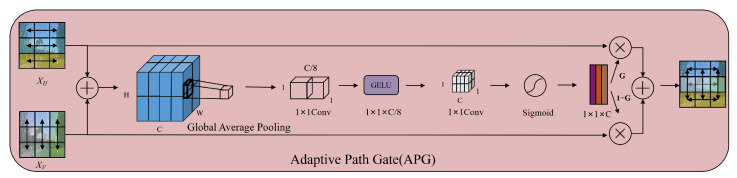
Illustration of the Adaptive Path Gating (APG) module.

In order to avoid the redundancy problems of features and information conflicts that are inevitable when initially adding axial features $X_{H}$ and $X_{V}$, SASFB has added an adaptive path gate APG mechanism. The module in Figure 4 uses a channel-wise attention strategy to dynamically adjust the contribution ratio of each axis, which can make the directional information more accurate and achieve adaptive fusion.

When the APG unit operates, it first sums $X_{H}$ and $X_{V}$ at the element level to initiate the feature integration step, and then uses global average pooling, also known as GAP, to compress spatial information into the C-dimensional channel descriptor *z*. Subsequently, a two-layer multilayer perceptron, also known as MLP, will be used to process this descriptor. First, a compression layer with ReLU activation will pass through, followed by an expansion layer with Sigmoid activation. Finally, the channel dimension gate vector *G* will be obtained, which has dimensions of $R^{B\times C\times 1\times 1}$. This *G* is actually a global context encoder that can capture the overall feature distribution of the current image and use it to strictly control the entire fusion process. We first use *G* to adjust $X_{H}$, then we use complementary weights 1−G to scale $X_{V}$. The two weighted features are then added to the original input *X* through a residual connection to obtain the fused representation $X_{fuse}$. This complementary weighting strategy allows the model to selectively emphasize direction-related features in a channel-wise manner.

(13) $$X_{fuse}=X+G⊙X_{H}+\left(1-G\right)⊙X_{V}$$

When rain marks are spread mostly horizontally, vertical axis scanning becomes particularly important in capturing such horizontal information clues. The APG mechanism will assign higher weights to $X_{V}$, and the corresponding value *G* will be smaller. This logic also holds true for vertical stripes, which can be achieved through the complementary weighting strategy mentioned in Equation (13). Afterwards, SASFB had the ability to choose combinations. Feature integration will always pay attention to the input content, which can improve the stability of the model when dealing with complex rain pattern shapes.

#### 2.3.3. Global Residual Connection and FFN Architecture of SASFB

In order to better express the content of the model, the fused feature representation $X_{fuse}$ is first normalized, and then passed over to a dedicated feedforward network for nonlinear channel-wise transformation, namely FFN, for further processing. In the network structure, 1×1 convolution is used to first expand the number of channels and then compress them back, while activation functions such as GELU are added to help abstract features more accurately. The SASFB module relies on global residual connections to integrate, combining the output of the final FFN with the initial raw input *X*. This design ensures smooth information propagation and facilitates the training stability essential for deep network optimization.

(14) $$X_{out}=X+\mathrm{FFN}\left(\mathrm{LN}\left(X_{fuse}\right)\right)$$

## 3. Experiments and Analysis

### 3.1. Experimental Setting

#### 3.1.1. Datasets

To evaluate the ability of the proposed method to remove synthetic rain streaks, we trained the network using the Rain13K dataset. This dataset was initially compiled on MPRNet [16]. We first integrated multiple rain scene datasets into this comprehensive dataset, such as Rain100H [11], Rain100L [11], Rain800 [52], Rain12000 [25], Rain14000 [10], and Rain12 [22], to cover different densities and directions of rain. To see if our model can be used in UAV-assisted perception scenarios, we specifically created a test set called RainVisDrone [45], which was obtained from VisDrone and used for subsequent object detection evaluation. We add simulated rainfall effects of different intensities to the drone images in the VisDrone validation set, so as to obtain three sub-datasets corresponding to light rain, moderate rain, and heavy rain. First, take the YOLOv8s detector trained with the original VisDrone data, and then use it to see if it can improve the detection effect of airborne targets on rainy days. We examine the methods mentioned in this evaluation plan from the perspectives of image restoration and drone perception.

#### 3.1.2. Baselines

In order to verify the effectiveness of our proposed method through experiments, we tested it on a synthesized dataset and compared it with twelve representative single image rain removal algorithms for benchmark testing. DerainNet [53], SEMI [54], DIDMDN [25], MSPFN [55], UMRL [56], MPRNet [32], RESCAN [57], PReNet [26], Restormer [27], FourierMamba [43], TAMambaIR [44] and TransMamba [42] were used as comparison benchmarks, and their corresponding quantitative test results are listed in Table 1. In some tables, the best-performing results are marked in bold, and the second-best results are marked with underlining. In order to make the comparison more fair and rigorous, the performance indicators of those early methods were directly quoted from their original published papers. We retrained and tested SFQMamba using the official code of recently competitive baseline models such as Restormer [27], FourierMamba [43], TAMambaIR [44], and TransMamba [42]. The experimental setup and data processing flow were completely consistent with our own proposed SFQMamba.

#### 3.1.3. Quantitative Evaluation

To quantitatively assess restoration quality, we employ the Peak Signal-to-Noise Ratio (PSNR) and the Structural Similarity Index Measure (SSIM) [58] as primary evaluation metrics. Specifically, PSNR measures the pixel-level reconstruction error between the restored image and the corresponding ground-truth image, and is defined as

(15) $$\mathrm{PSNR}\left(X,Y\right)=10\log_{10}\left(\frac{255^{2}}{\frac{1}{N}\sum_{n=1}^{N}{\left(x_{n}-y_{n}\right)}^{2}}\right),$$

where *X* and *Y* denote the restored image and the ground-truth image, respectively. $x_{n}$ and $y_{n}$ represent the intensity values of the *n*-th pixel in *X* and *Y*, and *N* is the total number of pixels. Since the images are represented in 8-bit format, the maximum possible pixel value is set to 255. A higher PSNR value indicates a lower reconstruction error and better image fidelity.

In contrast, SSIM evaluates perceptual similarity by comparing luminance, contrast, and structural information between the restored and ground-truth images. It is defined as

(16) $$\mathrm{SSIM}\left(X,Y\right)=\frac{\left(2\mu_{X}\mu_{Y}+C_{1}\right)\left(2\sigma_{XY}+C_{2}\right)}{\left(\mu_{X}^{2}+\mu_{Y}^{2}+C_{1}\right)\left(\sigma_{X}^{2}+\sigma_{Y}^{2}+C_{2}\right)},$$

where $\mu_{X}$ and $\mu_{Y}$ denote the mean intensity values of *X* and *Y*, respectively; $\sigma_{X}^{2}$ and $\sigma_{Y}^{2}$ denote their variances; and $\sigma_{XY}$ represents their covariance. $C_{1}$ and $C_{2}$ are small constants that are used to avoid numerical instability. A higher SSIM value indicates stronger structural similarity between the restored image and the ground truth.

### 3.2. Implementation Details

The training and testing programs were all conducted on the NVIDIA GeForce RTX 3090 graphics card. The basic channel dimension of the network was set to 32, and the overall structure consisted of four different feature extraction stages with 2, 3, 4, and 6 blocks, respectively. In addition, the expansion factor of the feedforward network was fixed at 2.0. During the 300,000 iterations of model training, the AdamW algorithm was used for model optimization. The momentum related parameter $\beta_{1}$ was set at 0.9, $\beta_{2}$ was 0.999, and the weight decay value was $1\times 10^{-4}$. We initially set the learning rate at $3\times 10^{-4}$, and then adjusted it by using cosine annealing to restart the cycle, gradually decreasing it to a minimum of $1\times 10^{-6}$. We used geometric transformations such as random rotation and horizontal flipping to expand the training data. During training, the batch size was set to 4, and the input patch size was 128×128. To speed up the calculation speed, automatic mixed precision, also known as AMP, was used throughout the training process. When optimizing, the reconstruction loss and the spectral coherence loss were combined as the goal.

### 3.3. Comparative Experiments on Synthetic Rain Datasets

We compare SFQMamba with representative deraining methods, including classical CNN-based models, Transformer-based models, and recent Mamba-related baselines. To reflect recent progress in this field, FourierMamba and TAMambaIR are newly added to the comparison. The quantitative results are shown in Table 1.

As shown in Table 1, SFQMamba achieves the best average performance, with 34.21 dB PSNR and 0.941 SSIM. Compared with TransMamba, which is the strongest competing method in terms of average performance, SFQMamba improves the average PSNR from 34.09 dB to 34.21 dB and slightly improves SSIM from 0.940 to 0.941. Although the improvement is modest and the visual differences between the two methods are not always very noticeable, SFQMamba demonstrates a more balanced performance on various rain image benchmarks. This suggests that the proposed spatial–frequency fusion and four-directional selectivity modeling method can produce further improvements compared to the TransMamba design.

**Figure 5:**
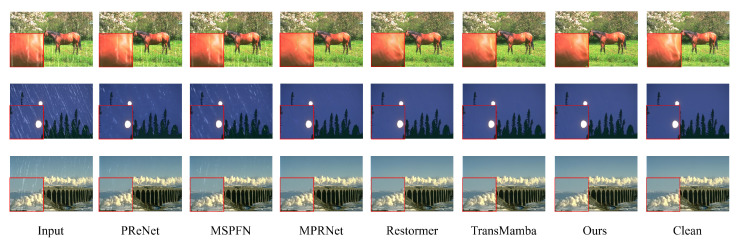
Visual comparison of different algorithms on the synthetic Rain100L dataset.

TransMamba remains highly competitive and performs well in Test100 and Test2800. However, SFQMamba achieves better results in Rain100H, Rain100L, and Test1200, and reaches the highest average score in all benchmarks, which is also consistent with the visual comparison in Figure 5. In particular, the improvement on Rain100L and Test1200 suggests that the frequency modulation branch is helpful for suppressing rain-related spectral interference, while the four-directional selective scanning mechanism better captures rain streaks with different orientations. Compared with FourierMamba and TAMambaIR, SFQMamba also shows higher average PSNR and SSIM, indicating more stable performance across different rain distributions.

In general, SFQMamba does not aim to replace TransMamba by a large margin in every individual case. Its main advantage lies in combining explicit spatial–frequency enhancement with four-directional state-space modeling, which leads to more balanced performance across different rain densities and streak distributions.

### 3.4. Ablation Studies

#### 3.4.1. Effectiveness Analysis of Dual-Branch Architecture

To validate our core motivation of reconciling global dependency modeling and local detail preservation, we conduct an ablation study on the macroscopic architecture. We define the Mamba-based pathway as the Global Branch and the CNN-based spatial–frequency pathway as the Local Branch. We evaluate three baseline variants: (1) w/o Local Branch: removing the CNN branch to rely solely on global state-space modeling; (2) w/o Global Branch: removing the Mamba branch to rely entirely on local convolutions and frequency modulation; and (3) w/o Hierarchical Interaction: retaining both branches but removing the progressive feature concatenation at each scale, replacing it with a simple late-fusion operation at the end of the network. As shown in Table 2, relying exclusively on the Global Branch or the Local Branch leads to a noticeable performance drop. This confirms that single-stream architectures cannot simultaneously handle long-range rain streaks and fine background textures. Furthermore, the degraded performance of the w/o Hierarchical Interaction variant demonstrates that our hierarchical interaction mechanism is crucial for effectively blending multi-scale local details with global context at different network depths.

#### 3.4.2. Effectiveness Analysis of Block

To analyze the contribution of each component, we conducted ablation experiments on the Rain100L test set. As shown in Table 3, the baseline model is built with NAFBlock as the CNN layer, CBSM as the Mamba-based modeling module, and simple addition as the fusion strategy. FreMLP and CA are not included in the baseline.

Variants a and b are used to evaluate the individual effects of FreMLP and CA in the CNN branch, respectively, while variant c combines both modules. Since variant c achieves better overall performance than the baseline, we keep FreMLP and CA enabled in the following variants to provide a fixed enhanced CNN branch. Based on this, variant d replaces CBSM with the proposed 4DSS to evaluate the effect of four-directional selective scanning, and variant e replaces the simple addition with APG to examine the effect of adaptive path fusion. The variant f integrates FreMLP, CA, 4DSS, and APG, corresponding to the full SFQMamba model.

#### 3.4.3. Effectiveness Analysis of Fused Enhance Block

From the aforementioned results, it is evident that the introduction of FreMLP for the global modulation of frequency domain amplitude components effectively supplements missing high-frequency details and corrects global structural distortions. Upon integrating FreMLP into the baseline network, the PSNR and SSIM metrics improved to 33.96 dB and 0.934 in Table 3 Variants c, respectively. Furthermore, the use of learnable parameters β and γ facilitates the dynamic balance of contributions of spatial features $Z_{spatial}$ and frequency features $X_{freq}$.

As indicated in Table 4, when the parameter β or γ is missed, both PSNR and SSIM decrease. This multiplicative guidance mechanism leverages global spectral statistics to selectively enhance or suppress the relative weighting of spatial and frequency features, thereby improving the model’s adaptability to varying degrees of degradation.

**Figure 6:**
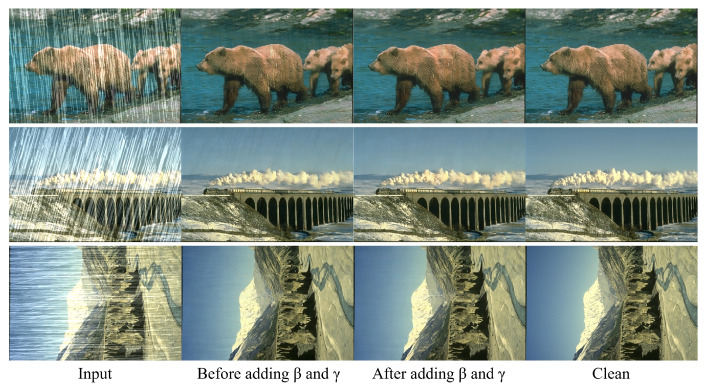
Visualization of robustness experimental results for learnable parameters on the synthetic Rain100H dataset.

As illustrated in Figure 5 and Figure 6, the Rain100L and Rain100H datasets were employed to represent rain streaks of varying thickness and density. The sparse rain streaks in Rain100L manifest primarily as low-frequency components in specific directions, whereas the dense heavy rain in Rain100H is characterized by a wide-area high-frequency distribution on a cross-scale. As shown in Table 5, the multiplicative guidance mechanism proposed in this paper leverages spectral statistical properties to adaptively reinforce the suppression of high-frequency noise in heavy rain scenarios, while optimizing the preservation of texture details by the spatial branch in light rain conditions.

By introducing β and γ to dynamically regulate feature extraction in both spatial and frequency domains, the model demonstrates enhanced robustness when processing the distinct characteristics of the Rain100L and Rain100H datasets.

Furthermore, as indicated in Table 3, the integration of a Channel Attention (CA) module into the spatial branch re-weights feature channels to explicitly heighten the response to regions with residual rain. This enables the network to focus more effectively on key features for deraining, further increasing performance metrics to 33.91 dB and 0.933.

#### 3.4.4. Effectiveness Analysis of Spatial-Aware Selective Fusion Block

The SASFB module is designed to address the inadequacies of traditional 1D scanning strategies in modeling rain streaks at arbitrary angles, as well as the problem of non-adaptive scanning feature fusion.

(1) Effectiveness Analysis of 4DSS. The traditional Mamba architecture that we often see uses cascaded bidirectional SSM modules, also known as CBSM, but these modules cannot fully capture long-distance dependencies on diagonal and horizontal paths. We replaced the original CBSM layer with the 4DSS module, while retaining the simplest element-wise addition fusion method. The final measured PSNR and SSIM increased to 34.07 dB and 0.939, respectively. This actually indicates that the 4DSS mechanism can effectively broaden the receptive field of the SSMs, covering all major directions in the 2D plane, which is particularly crucial for simulating complex geometric shapes such as rain streaks.

(2) Effectiveness Analysis of the Adaptive Path Gating (APG) Mechanism. After obtaining complementary axial features $X_{H}$ and $X_{V}$, simply adding them together can easily lead to redundancy and conflict of characteristics. To solve this problem, we used the APG mechanism. This mechanism dynamically calculates the gate control weight *G* through channel attention and uses it for complementary weighted fusion. After using APG, the model achieved optimal performance, with a peak signal-to-noise ratio (PSNR) of 34.17 decibels and a structural similarity SSIM of 0.940. APG can bring many optimizations, which can be seen that it endows the model with strong capabilities, not only focusing on content, but also selectively integrating. It enables the dynamic adjustment of contributions from horizontal and vertical axis features according to the actual geometric distribution of rain streaks, thereby significantly bolstering robustness against complex rain streak geometries.

The experimental results discussed above demonstrate the validity of the distinct modules proposed in our algorithm, including multi-scale spatial modeling, Frequency-domain Modulation Enhancement (FreMLP), learnable residual coupling (β, γ), 4DSS, and APG, in enhancing image deraining performance. These findings provide a theoretical foundation for further research and optimization of the proposed network.

### 3.5. Application-Oriented Evaluation for UAV Secure Communication in IoT Scenarios

This experiment is intended to evaluate whether SFQMamba can serve as a visual enhancement front-end for UAV-oriented perception, rather than to claim that the network is exclusively designed for UAV images. To verify whether the proposed deraining model improves downstream UAV perception, we perform an object detection evaluation on a VisDrone-derived RainVisDrone test set using a VisDrone-trained YOLOv8s detector. In the full validation split, rainy degradation consistently reduces detection accuracy. The clean images obtain an AP of 0.2482, an AP${⁠}_{50}$ of 0.4141, and an AP${⁠}_{75}$ of 0.2532. Under light, medium, and heavy rain, the AP decreases to 0.2283, 0.1998, and 0.1705, respectively, while the AP${⁠}_{50}$ decreases to 0.3823, 0.3402, and 0.2965.

**Figure 7:**
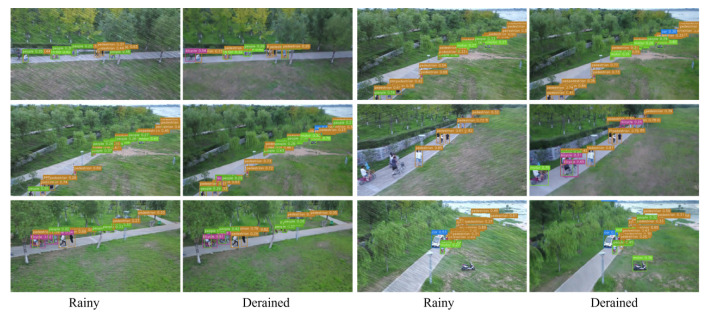
Visualization of the results from the UAV target detection experiment.

After applying the proposed deraining model to the medium-rain UAV subset, the AP increases from 0.1267 to 0.2003, the AP${⁠}_{50}$ increases from 0.2399 to 0.3460, and the AP${⁠}_{75}$ increases from 0.1167 to 0.2063. The number of ground-truth-matched detections also increases from 114 to 131 at a confidence threshold of 0.25. Figure 7 shows a visualization of the results of this experiment. These results demonstrate that the proposed deraining model can effectively improve the detection of downstream UAV objects under rainy conditions.

This experiment is an application-oriented offline evaluation rather than an onboard UAV deployment test. All results are obtained on a workstation GPU, which is different from embedded UAV hardware. Thus, the reported improvement mainly indicates that deraining can benefit downstream UAV perception under rainy conditions, while real-time deployment performance on edge UAV devices remains to be evaluated in future work.

## 4. Conclusions

In this work, we propose a dual-stream deraining network based on spatial–frequency collaboration to address the trade-off between local detail restoration and long-range dependency modeling. The network contains two complementary branches. The Spatial–Frequency Collaborative Enhancement Branch uses an efficient spatial foundation and introduces a Frequency-domain Modulation Path to fuse FFT-based global structural priors with local spatial features. Meanwhile, the Spatial-Aware Selective Branch employs a Four-Directional Selective Scanning mechanism to overcome the one-dimensional modeling limitation of conventional State Space Models, enabling more effective representation of complex rain streaks and background structures in two-dimensional images.

Experimental results show that the proposed architecture achieves strong reconstruction performance on synthetic rain benchmarks while maintaining efficient global modeling. In addition, the VisDrone-derived RainVisDrone evaluation shows that the restored images improve the detection of YOLOv8s-based UAV objects under medium-rain conditions, with AP, AP${⁠}_{50}$, and AP${⁠}_{75}$ increasing by 0.0737, 0.1060, and 0.0897, respectively, over rainy inputs. These results indicate that the proposed deraining model can improve both low-level image restoration and downstream UAV rainy-scene perception in IoT-oriented applications.

It should be noted that the UAV-oriented evaluation in this work is conducted in an offline workstation environment rather than on embedded UAV hardware. Although the dual-branch design improves restoration and perception performance, it also introduces additional computational overhead due to parallel feature extraction and feature fusion. Therefore, its real-time deployment efficiency on resource-constrained edge devices still requires further study. Future work will focus on lightweight model optimization, including knowledge distillation and weight pruning, and on evaluating the model in more diverse adverse weather conditions, such as rain, fog, and snow.

## Figures and Tables

**Table 1:** Comparative experimental results of different algorithms on synthetic rain datasets.

Methods	Test100		Rain100H		Rain100L		Test2800		Test1200		Average	
	PSNR	SSIM	PSNR	SSIM	PSNR	SSIM	PSNR	SSIM	PSNR	SSIM	PSNR	SSIM
DIDMDN [25]	22.56	0.818	17.35	0.524	25.23	0.741	28.13	0.867	29.65	0.901	24.58	0.770
PReNet [26]	24.81	0.851	26.77	0.858	32.44	0.950	31.75	0.916	31.36	0.911	29.42	0.897
Restormer [27]	32.00	0.923	**31.46**	0.904	38.99	0.978	**34.18**	0.944	33.19	0.926	33.96	0.935
MPRNet [32]	30.27	0.897	30.41	0.890	36.40	0.965	33.64	0.938	32.91	0.916	32.73	0.921
TransMamba [42]	32.35	0.941	31.01	0.902	39.31	0.970	33.91	**0.950**	33.87	0.938	34.09	0.940
FourierMamba [43]	**32.41**	**0.943**	31.11	0.902	39.27	0.975	32.98	0.940	33.64	0.931	33.88	0.938
TAMambaIR [44]	32.28	0.940	31.27	0.903	39.22	0.972	33.87	0.934	33.69	0.933	34.06	0.936
DerainNet [53]	22.77	0.810	14.92	0.592	27.03	0.884	24.31	0.861	23.38	0.835	22.48	0.796
SEMI [54]	22.35	0.788	16.56	0.486	25.03	0.842	24.43	0.782	26.05	0.822	22.88	0.744
MSPFN [55]	27.50	0.876	28.66	0.860	32.40	0.933	32.82	0.930	32.39	0.916	30.75	0.903
UMRL [56]	24.41	0.829	26.01	0.832	29.18	0.923	29.97	0.905	30.55	0.910	28.02	0.880
RESCAN [57]	25.00	0.835	26.36	0.786	29.80	0.881	31.29	0.904	30.51	0.882	28.59	0.857
Ours	32.31	0.929	31.35	**0.905**	**39.41**	**0.984**	34.02	0.947	**33.97**	**0.940**	**34.21**	**0.941**

**Table 2:** Ablation study on the effectiveness of the dual-branch architecture on the Rain100L test set.

	w/o Local Branch	w/o Global Branch	w/o Hierarchical Interaction	SFQMamba
PSNR (dB)	33.67	33.88	33.98	**34.17**
SSIM	0.933	0.936	0.938	**0.940**

**Table 3:** Ablation study of different components on the Rain100L test set.

Variants		Baseline	a	b	c	d	e	f
CNN Layer	FreMLP	✕	✓	✕	✓	✓	✓	✓
	CA	✕	✕	✓	✓	✓	✓	✓
Mamba Layer	CBSM [42]	✓	✓	✓	✓	✕	✓	✕
	4DSS	✕	✕	✕	✕	✓	✕	✓
	Addition	✓	✓	✓	✓	✓	✕	✕
	APG	✕	✕	✕	✕	✕	✓	✓
PSNR (dB)		33.87	33.92	33.89	33.96	34.07	34.12	**34.17**
SSIM		0.930	0.932	0.929	0.934	0.937	0.939	**0.940**

**Table 4:** Comparative experimental results of incorporating learnable parameters.

Evaluation Metrics	*β*	*γ*	*β* & *γ*
PSNR (dB)	33.74	33.76	**33.96**
SSIM	0.932	0.933	**0.934**

**Table 5:** Comparative analysis of the robustness effects of learnable parameters.

	Before Adding β and γ		After Adding β and γ	
Datasets	Rain100L	Rain100H	Rain100L	Rain100H
PSNR (dB)	33.72	29.75	33.89	30.26
SSIM	0.934	0.874	0.937	0.881

## Data Availability

Data supporting reported results can be found in the publicly available Rain13K and Raindrop datasets.

## References

[1] Kang L.-W., Lin C.-W., Lin C.-T., Lin Y.-C. (2012). Self-Learning-Based Rain Streak Removal for Image/Video. 2012 IEEE International Symposium on Circuits and Systems (ISCAS).

[2] Luo Y., Xu Y., Ji H. (2015). Removing Rain from a Single Image via Discriminative Sparse Coding. Proceedings of the IEEE International Conference on Computer Vision (ICCV).

[3] Qian R., Tan R.T., Yang W., Su J., Liu J. (2018). Attentive Generative Adversarial Networks for Raindrop Removal from a Single Image. Proceedings of the IEEE Conference on Computer Vision and Pattern Recognition (CVPR).

[4] Hu J., Li J., Hou Z., Jiang J., Liu C., Zhang Y. (2023). Potential Auto-Driving Threat: Universal Rain-Removal Attack. arXiv.

[5] Xu N., Yang J.J. (2024). Leveraging Scene Geometry and Depth Information for Robust Image Deraining. Computers.

[6] Yuan Y., Yang W., Ren W., Liu J., Scheirer W.J., Wang Z. (2019). UG2+ Track 2: A Collective Benchmark Effort for Evaluating and Advancing Image Understanding in Poor Visibility Environments. Proceedings of the IEEE/CVF Conference on Computer Vision and Pattern Recognition Workshops (CVPRW).

[7] Fan C.-M., Liu T.-J., Liu K.-H. (2023). Compound Multi-Branch Feature Fusion for Image Deraindrop. Proceedings of the IEEE International Conference on Image Processing (ICIP).

[8] Li S., Araujo I.B., Ren W., Wang Z., Tokuda E.K., Junior R.H., Cesar-Junior R., Zhang J., Guo X., Cao X. (2019). Single Image Deraining: A Comprehensive Benchmark Analysis. Proceedings of the IEEE/CVF Conference on Computer Vision and Pattern Recognition (CVPR).

[9] Shao M.-W., Li L., Meng D.-Y., Zuo W.-M. (2021). Uncertainty Guided Multi-Scale Attention Network for Raindrop Removal from a Single Image. IEEE Transactions on Image Processing.

[10] Fu X., Huang J., Zeng D., Huang Y., Ding X., Paisley J. (2017). Removing Rain from Single Images via a Deep Detail Network. Proceedings of the IEEE Conference on Computer Vision and Pattern Recognition (CVPR).

[11] Yang W., Tan R.T., Feng J., Liu J., Guo Z., Yan S. (2017). Deep Joint Rain Detection and Removal from a Single Image. Proceedings of the IEEE Conference on Computer Vision and Pattern Recognition (CVPR).

[12] Wang T., Yang X., Xu K., Chen S., Zhang Q., Lau R.W. (2019). Spatial Attentive Single-Image Deraining with a High Quality Real Rain Dataset. Proceedings of the IEEE/CVF Conference on Computer Vision and Pattern Recognition (CVPR).

[13] Zhang K., Li D., Luo W., Ren W. (2021). Dual Attention-in-Attention Model for Joint Rain Streak and Raindrop Removal. IEEE Trans. Image Process..

[14] Wang H., Xie Q., Zhao Q., Meng D. (2020). A Model-Driven Deep Neural Network for Single Image Rain Removal. Proceedings of the IEEE/CVF Conference on Computer Vision and Pattern Recognition (CVPR).

[15] Zhang G., Wu Q., Cui M., Zhang R. (2019). Securing UAV Communications via Joint Trajectory and Power Control. IEEE Trans. Wirel. Commun..

[16] Li S., Duo B., Di Renzo M., Tao M., Yuan X. (2021). Robust Secure UAV Communications with the Aid of Reconfigurable Intelligent Surfaces. IEEE Trans. Wirel. Commun..

[17] Tatar Mamaghani M., Zhou X., Yang N., Swindlehurst A.L. (2023). Secure Short-Packet Communications via UAV-Enabled Mobile Relaying: Joint Resource Optimization and 3D Trajectory Design. IEEE Trans. Wirel. Commun..

[18] Liu X., Suganuma M., Sun Z., Okatani T. (2019). Dual Residual Networks Leveraging the Potential of Paired Operations for Image Restoration. Proceedings of the IEEE/CVF Conference on Computer Vision and Pattern Recognition (CVPR).

[19] Chen H., Wang Y., Guo T., Xu C., Deng Y., Liu Z., Ma S., Xu C., Xu C., Gao W. (2021). Pre-Trained Image Processing Transformer. Proceedings of the IEEE/CVF Conference on Computer Vision and Pattern Recognition (CVPR).

[20] Chen L., Lu X., Zhang J., Chu X., Chen C. (2021). HINet: Half Instance Normalization Network for Image Restoration. Proceedings of the IEEE/CVF Conference on Computer Vision and Pattern Recognition Workshops (CVPRW).

[21] Chang Y., Yan L., Zhong S. (2017). Transformed Low-Rank Model for Line Pattern Noise Removal. Proceedings of the IEEE International Conference on Computer Vision (ICCV).

[22] Li Y., Tan R.T., Guo X., Lu J., Brown M.S. (2016). Rain Streak Removal Using Layer Priors. Proceedings of the IEEE Conference on Computer Vision and Pattern Recognition (CVPR).

[23] Chen X., Li H., Li M., Pan J. (2023). Learning a Sparse Transformer Network for Effective Image Deraining. Proceedings of the IEEE/CVF Conference on Computer Vision and Pattern Recognition (CVPR).

[24] Potlapalli V., Zamir S.W., Khan S., Khan F.S. PromptIR: Prompting for All-in-One Blind Image Restoration. Proceedings of the Advances in Neural Information Processing Systems (NeurIPS).

[25] Zhang H., Patel V.M. (2018). Density-Aware Single Image De-Raining Using a Multi-Stream Dense Network. Proceedings of the IEEE Conference on Computer Vision and Pattern Recognition (CVPR).

[26] Ren D., Zuo W., Hu Q., Zhu P., Meng D. (2019). Progressive Image Deraining Networks: A Better and Simpler Baseline. Proceedings of the IEEE/CVF Conference on Computer Vision and Pattern Recognition (CVPR).

[27] Zamir S.W., Arora A., Khan S., Hayat M., Khan F.S., Yang M.-H. (2022). Restormer: Efficient Transformer for High-Resolution Image Restoration. Proceedings of the IEEE/CVF Conference on Computer Vision and Pattern Recognition (CVPR).

[28] Wang Z., Cun X., Bao J., Zhou W., Liu J., Li H. (2022). Uformer: A General U-Shaped Transformer for Image Restoration. Proceedings of the IEEE/CVF Conference on Computer Vision and Pattern Recognition (CVPR).

[29] Xiao J., Fu X., Liu A., Wu F., Zha Z.-J. (2022). Image De-Raining Transformer. IEEE Trans. Pattern Anal. Mach. Intell..

[30] Lim B., Son S., Kim H., Nah S., Lee K.M. (2017). Enhanced Deep Residual Networks for Single Image Super-Resolution. Proceedings of the IEEE Conference on Computer Vision and Pattern Recognition Workshops (CVPRW).

[31] Zhang K., Zuo W., Chen Y., Meng D., Zhang L. (2017). Beyond a Gaussian Denoiser: Residual Learning of Deep CNN for Image Denoising. IEEE Trans. Image Process..

[32] Zamir S.W., Arora A., Khan S., Hayat M., Khan F.S., Yang M.-H., Shao L. (2021). Multi-Stage Progressive Image Restoration. Proceedings of the IEEE/CVF Conference on Computer Vision and Pattern Recognition (CVPR).

[33] Vaswani A., Shazeer N., Parmar N., Uszkoreit J., Jones L., Gomez A.N., Kaiser L., Polosukhin I. Attention Is All You Need. Proceedings of the Advances in Neural Information Processing Systems (NeurIPS).

[34] Liu Z., Lin Y., Cao Y., Hu H., Wei Y., Zhang Z., Lin S., Guo B. (2021). Swin Transformer: Hierarchical Vision Transformer Using Shifted Windows. Proceedings of the IEEE/CVF International Conference on Computer Vision (ICCV).

[35] Li B., Liu X., Hu P., Wu Z., Lv J., Peng X. (2022). All-in-One Image Restoration for Unknown Corruption. Proceedings of the IEEE/CVF Conference on Computer Vision and Pattern Recognition (CVPR).

[36] Liang J., Cao J., Sun G., Zhang K., Van Gool L., Timofte R. (2021). SwinIR: Image Restoration Using Swin Transformer. Proceedings of the IEEE/CVF International Conference on Computer Vision (ICCV).

[37] Tu Z., Talebi H., Zhang H., Yang F., Milanfar P., Bovik A., Li Y. (2022). MAXIM: Multi-Axis MLP for Image Processing. Proceedings of the IEEE/CVF Conference on Computer Vision and Pattern Recognition (CVPR).

[38] Gu A., Goel K., Ré C. (2021). Efficiently Modeling Long Sequences with Structured State Spaces. arXiv.

[39] Gu A., Dao T. (2023). Mamba: Linear-Time Sequence Modeling with Selective State Spaces. arXiv.

[40] Guo H., Li J., Dai T., Ouyang Z., Ren X., Xia S.-T. (2024). MambaIR: A Simple Baseline for Image Restoration with State-Space Model. Proceedings of the European Conference on Computer Vision (ECCV).

[41] Zhu L., Liao B., Zhang Q., Wang X., Liu W., Wang X. (2024). Vision Mamba: Efficient Visual Representation Learning with Bidirectional State Space Model. arXiv.

[42] Sun S., Ren W., Zhou J., Gan J., Wang R., Cao X. (2024). A Hybrid Transformer–Mamba Network for Single Image Deraining. arXiv.

[43] Li D., Liu Y., Fu X., Huang J., Xu S., Zhu Q., Zha Z.-J. FourierMamba: Fourier Learning Integration with State Space Models for Image Deraining. Proceedings of the International Conference on Learning Representations (ICLR).

[44] Peng L., Di X., Feng Z., Li W., Pei R., Wang Y., Fu X., Cao Y., Zha Z.-J. Directing Mamba to Complex Textures: An Efficient Texture-Aware State Space Model for Image Restoration. Proceedings of the Thirty-Fourth International Joint Conference on Artificial Intelligence (IJCAI).

[45] Xi Y., Jia W., Miao Q., Feng J., Liu X., Li F. (2023). CoDerainNet: Collaborative Deraining Network for Drone-View Object Detection in Rainy Weather Conditions. Remote Sens..

[46] Jacobsen J.-H., Smeulders A., Oyallon E. i-RevNet: Deep Invertible Networks. Proceedings of the International Conference on Learning Representations (ICLR).

[47] Shi W., Caballero J., Huszár F., Totz J., Aitken A.P., Bishop R., Rueckert D., Wang Z. (2016). Real-Time Single Image and Video Super-Resolution Using an Efficient Sub-Pixel Convolutional Neural Network. Proceedings of the IEEE Conference on Computer Vision and Pattern Recognition (CVPR).

[48] Ba J.L., Kiros J.R., Hinton G.E. (2016). Layer Normalization. arXiv.

[49] Yu F., Koltun V. (2015). Multi-Scale Context Aggregation by Dilated Convolutions. arXiv.

[50] Chen L., Chu X., Zhang X., Sun J. (2022). Simple Baselines for Image Restoration. Proceedings of the European Conference on Computer Vision (ECCV).

[51] Hu J., Shen L., Sun G. (2018). Squeeze-and-Excitation Networks. Proceedings of the IEEE Conference on Computer Vision and Pattern Recognition (CVPR).

[52] Zhang H., Sindagi V., Patel V.M. (2019). Image De-Raining Using a Conditional Generative Adversarial Network. IEEE Trans. Circuits Syst. Video Technol..

[53] Fu X., Huang J., Ding X., Liao Y., Paisley J. (2017). Clearing the Skies: A Deep Network Architecture for Single-Image Rain Removal. IEEE Trans. Image Process..

[54] Wei W., Meng D., Zhao Q., Xu Z., Wu Y. (2019). Semi-Supervised Transfer Learning for Image Rain Removal. Proceedings of the IEEE/CVF Conference on Computer Vision and Pattern Recognition (CVPR).

[55] Jiang K., Wang Z., Yi P., Chen C., Han Z., Lu T., Ma J. (2020). Multi-Scale Progressive Fusion Network for Single Image Deraining. Proceedings of the IEEE/CVF Conference on Computer Vision and Pattern Recognition (CVPR).

[56] Yasarla R., Patel V.M. (2019). Uncertainty Guided Multi-Scale Residual Learning Using a Cycle Spinning CNN for Single Image De-Raining. Proceedings of the IEEE/CVF Conference on Computer Vision and Pattern Recognition (CVPR).

[57] Li X., Wu J., Lin Z., Liu H., Zha H. (2018). Recurrent Squeeze-and-Excitation Context Aggregation Net for Single Image Deraining. Proceedings of the European Conference on Computer Vision (ECCV).

[58] Wang Z., Bovik A.C., Sheikh H.R., Simoncelli E.P. (2004). Image Quality Assessment: From Error Visibility to Structural Similarity. IEEE Trans. Image Process..

